# Acupuncture combined with opioid for treatment of lung cancer-related pain: A systematic review and meta-analysis

**DOI:** 10.1097/MD.0000000000040158

**Published:** 2024-10-18

**Authors:** Liting Jia, Keyi Wang, Shuquan Chen

**Affiliations:** a Shandong University of Traditional Chinese Medicine, Jinan, Shandong Province, China; b Jinan Integrated Traditional Chinese and Western Medicine Hospital, Jinan, Shandong Province, China.

**Keywords:** acupuncture, lung cancer, meta-analysis, opioids, pain

## Abstract

**Background:**

Many individuals diagnosed with lung cancer suffer from tremendous pain, and it is crucial to implement more effective measures to assist these patients in alleviating their pain. The present study utilizes a meta-analysis to evaluate the safety and efficacy of acupuncture combined with opioids for treating lung cancer-related pain in patients.

**Methods:**

We have searched 8 electronic databases: The Cochrane Library, PubMed, Embase, Web of Science, China National Knowledge Infrastructure, China Science and Technology Journal Database, Wanfang Database, and SinoMed. We included all randomized controlled trials of acupuncture combined with opioids for lung cancer-related pain in adults. We observed the main outcome indicators, including pain relief rates, numeric rating scale scores, and adverse events. Two researchers independently conducted literature screening, literature data extraction, and assessment of bias risk in the literature quality. Any disagreements were resolved through discussions between the 2 researchers or consultations with a third researcher. The risk of bias in the included studies was assessed using the revised risk of bias assessment tool. The overall quality of evidence for each outcome was evaluated using Grading of Recommendations, Assessment, Development and Evaluations.

**Results:**

We retrieved 812 lung cancer patients from 11 trials. The study showed that compared to opioids alone, the combination of acupuncture and opioids significantly reduced numeric rating scale scores, increased pain relief rates, and decreased the occurrence of side effects.

**Conclusion:**

The current evidence indicates that combining acupuncture with opioid analgesics is superior to using opioid analgesics alone for managing lung cancer-related pain. Additionally, this combination therapy has fewer adverse reactions.

## 1. Introduction

Pain is one of the most painful symptoms among lung cancer patients. There are 38% of patients who experience moderate and severe pain in lung cancer.^[1]^ As the disease progresses, the quality of life for lung cancer patients will continue to decline, with symptoms deteriorating noticeably. Pain accounts for 59% of these symptoms.^[2]^ Despite the increased focus on cancer prevention and treatment, the management of cancer-related pain has largely remained unchanged. At present, opioids are still the mainstay for treating lung tumor patients with moderate to severe pain, and long-term use of opioid analgesics has more adverse effects.^[3]^ While opioids have been effective in managing pain for cancer patients, concerns regarding their addictive potential and adverse effects have increased in recent years. Moreover, research has proven that a significant proportion of patients with tumors continue to experience inadequate pain relief.^[4]^

A European survey discovered that more than 30% of patients experiencing cancer pain choose complementary or supplementary treatments, such as acupuncture, to alleviate their pain. As the population continues to grow and age, it is expected that the number of individuals seeking alternative treatments will increase annually.^[5]^ To address these concerns, the US National Comprehensive Cancer Network has included acupuncture therapy as a comprehensive intervention for cancer pain in their guidelines for adult patients.^[6]^ This recognition highlights the increasing acceptance of acupuncture as a beneficial treatment for pain management in cancer patients. By incorporating acupuncture into cancer pain management strategies, healthcare professionals can offer patients a non-pharmacological option that has shown promise in alleviating pain and improving overall well-being. However, it is necessary to notice that acupuncture should not replace conventional treatments, but rather be used as an adjunct therapy to complement existing pain management approaches.

Acupuncture, as one of the supplementary therapies, is safe and effective in relieving cancer pain.^[7]^ As an adjuvant therapy in the treatment of cancer pain, acupuncture can not only reduce the degree of pain and the use of analgesic drugs, but also significantly improve the physical function of patients with cancer pain. It can help relieve anxiety, depression, and other negative emotions, thereby enhancing the quality of life and prognosis of the disease.^[8]^ Studies have revealed that electro-acupuncture significantly reduces pain in animal models.^[9]^ With the increasing number of clinical trials examining the efficacy of acupuncture in treating cancer pain, this study adhered to the principles of evidence-based medicine and conducted a search for randomized controlled trials that met the specified inclusion criteria. To assess the analgesic impact of combining acupuncture with opioid analgesics for lung cancer-related pain, with the aim of offering a reference and basis for clinical decision-making.

## 2. Materials and methods

### 2.1. Research design

This study was conducted according to the Preferred Reporting Items for Systematic Reviews and Meta-Analyses guidelines.^[10]^ The review scheme of this study has been registered on PROSPERO, the international prospective register of systematic reviews (CRD42023401712).

### 2.2. Search strategy

The Electronic databases we have searched included The PubMed, The Cochrane Library, The Embase, Web of Science, China National Knowledge Infrastructure, China Science Technology Journal Database, Wanfang Database, and SinoMed Database. Studies included ranged from inception to February 2023 for randomized controlled trials (RCTs). The retrieved literature had no language restrictions. The complete retrieval strategy is presented as Appendix S1, Supplemental Digital Content, http://links.lww.com/MD/N764.

### 2.3. Inclusion criteria

The inclusion criteria were as follows: (1) types of studies: the included studies were all clinical RCTs, whether blinded or not. The published papers are mainly published in the form of papers and abstracts. (2) Types of participants: age range 18 to 85 years, with no gender restrictions, and expected survival of at least 1 month. Patients diagnosed with lung cancer by pathology and experiencing lung cancer-related pain typically report moderate to severe pain. Patients can clearly state their illness and pain to the medical staff and cooperate with them. (3) Types of interventions: the control group received opioid analgesics. The intervention group was based on the control group and combined with acupuncture treatment. Acupuncture therapy specifically includes: ordinary acupuncture, moxibustion, electric acupuncture, ear needle, fire needle, plum blossom needle, acupoint pressuring, acupoint application, acupoint embedding, acupoint injection, percutaneous acupoint electrical stimulation, and other treatment methods. (4) Types of outcome evaluations: considered all outcome reports in the included studies, and required that each study included at least 1 pain-related clinical indicator. The primary outcome measure was the pain intensity score (continuity variables, measured by numeric rating scale [NRS], visual analogue scale [VAS], and brief pain inventory [BPI]). The Secondary outcome measures included the analgesic response rate (dichotomy data, reported as valid and invalid) and adverse reactions. Adverse reactions mainly include nausea, vomiting, constipation, lethargy, and skin diseases (pruritus, rash, and urticaria).

### 2.4. Exclusion criteria

The exclusion criteria were as follows: (1) combined with other cancers. (2) combine pain in 1 or more other disease types. (3) The experimental design is not strict, and the experimental data are incomplete. (4) There are no clear outcome indicator. (5) Using inappropriate statistical methods. (6) The same study, repeated published literature. (7) The original text cannot obtained.

### 2.5. Literature screening and data extraction

Data were independently screened and extracted by 2 researchers, with a third researcher participating in the discussion and decision-making process in cases of disagreement. Duplicate documents were removed using the EndNote X9 literature management software. Read the titles and abstracts of the literature for preliminary screening, and then screen out the included literature based on the full text. The data from the literature were extracted and organized into an Excel table. This table includes basic information about the participants, research design data, specific intervention measures, and intervention outcomes.

### 2.6. Bias risk assessment

Two researchers independently assessed the quality of each included study using the Version 2 of the Cochrane tool for assessing risk of bias in randomized trials.^[11]^ The evaluation included the following criteria: randomization process, deviations from intended interventions, missing outcome data, measurement of the outcome, and selection of the reported results.

### 2.7. Evidence evaluation

We used the Grading of Recommendations, Assessment, Development and Evaluations (GRADEpro)^[12]^ to assess the quality of evidence for each outcome measure in this study. RCT trials are classified as high-quality evidence, and it is essential to assess whether the evidence needs to be downgraded based on 5 factors: the risk of bias, inconsistency, indirectness, imprecision, and publication bias. The final evidence quality is divided into 4 levels: “high,” “medium,” “low,” and “very low.”

### 2.8. Statistical method

#### 2.8.1. Comprehensive data

This study utilized RevMan 5.4 software for data merging and analysis. Binary variables were statistically described using the relative risk (RR). Continuous variables were described by the mean difference (MD). Each effect size was statistically analyzed using a 95% confidence interval (CI). The chi-square test was used to evaluate the heterogeneity between studies. A random-effects model was selected when I^2^ > 50%, indicating greater heterogeneity. Conversely, a fixed-effects model was chosen when I^2^ < 50%, indicating less heterogeneity. If there is significant heterogeneity, conduct subgroup analysis to identify the source of heterogeneity, and use sensitivity analysis to assess the stability of the results. The funnel plots were used to analyze and detect publication bias when a sufficient number of studies were included.

#### 2.8.2. Association rule mining

We first use Excel to complete the frequency statistics of acupoints, and then utilize SPSS Modeler 18.0 to analyze Apriori association rules and create complex network graphs.

## 3. Results

### 3.1. Research selection

**Figure 1.**
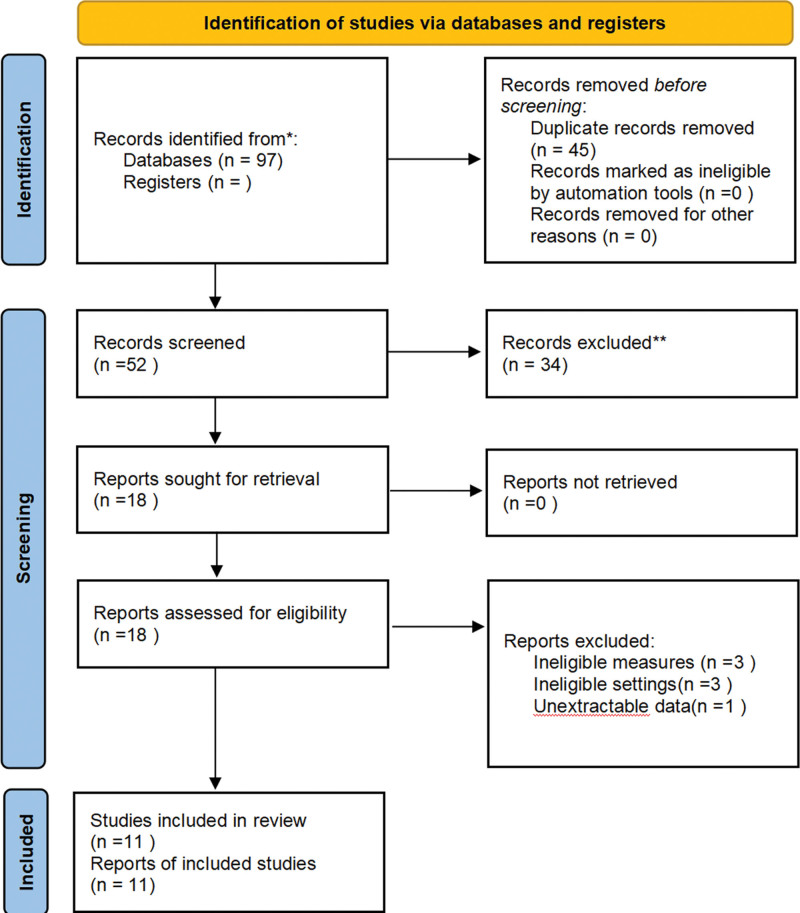
Flow diagram of literature screening.

A total of 97 articles were obtained after searching the database according to the designed retrieval strategy. There were 45 duplicate articles screened out using EndNote X9 software. After conducting an initial review of the title and abstract of the literature, 34 nonconforming articles were excluded. By carefully reading the full text, we finally included 11 qualified literature sources, basic information from the included studies was presented into a table. The basic characteristics of the study are shown in Table 1. All screening processes are detailed in Figure 1.

**Table 1:** Baseline characteristics of the included randomized controlled trials.

Study (author/year)	Sample size (T/C)	Intervention		Session frequency and duration	Main outcomes
		T	C		
WangYing (2017)^[12]^	30/30	EA + C	Oxycodone sustained-release tablets, q12 h	30 minutes qd 14 days	②③
WangHui (2018)^[13]^	30/30	EA + C	Oxycodone sustained-release tablets, q12 h	30 minutes qd 14 days	②③
WuSheng (2020)^[14]^	80/80	AT + AI + C	Oxycodone hydrochloride prolonged-release tablets 10–20 mg, q12 h	25–30 minutes qod 28 days	①③
LiuWeiji (2012)^[15]^	30/30	AT + C	Morphine hydrochloride injection im, 10 mg, q5 h	60 minutes bid 10 days	②④
ZhangHui (2016)^[16]^	39/39	TCMNI+ TEAS + C	Oxycodone and acetaminophen tablets, q6 h	30 minutes bid 28 days	①②
WangHong (2021)^[17]^	30/30	TEAS + C	Oxycodone and acetaminophen tablets, q12 h	30 minutes qd 28 days	③
ZhangLiwen (2018)^[18]^	30/30	WCBM + C	Morphine hydrochloride sustained-release tablets 10–20 mg, q12 h	30 minutes qod 7 days	①③
ZhangLiwen (2019)^[19]^	35/35	WCBM + C	Morphine hydrochloride sustained-release tablets 10–20 mg, q12 h	30 minutes qod 7 days	②
CuiFeng (2014)^[20]^	30/30	CMA + C	Tramadol hydrochloride sustained-release tablets 100 mg, q12 h	Not mentioned	①②③
NiJuan (2021)^[21]^	40/40	WA + APB + C	Codeine phosphate tablets 30–60 mg, q6–8 h	1 h qd 14 days	①②③
SongDongsheng (2015)^[22]^	32/32	ACE + AI + C	Morphine hydrochloride sustained-release tablets 10 mg, q12 h	10 days	①

① Analgesia effective rate, ② NRS: numerical rating scale, ③ adverse reactions, ④ VAS: visual analogue scale.

ACE = acupoint catgut embedding, AI = acupoint injection, APB = auricular acupoint pressing bean, AT = acupuncture, C = control group, CMA = Chinese Medicine Acupoint Application, EA = electro-acupuncture, MB = moxibustion, T = treatment group, TCMNI = traditional Chinese medical nursing intervention, TEAS = Transcutaneous Electrical Acupoint Stimulation, WA = wrist-ankle acupuncture, WCBM = warm compress back meridians.

### 3.2. Research description

A total of 812 patients with lung cancer were included in the 11 eligible articles. The experimental group and the control group consisted of 406 cases each. Baselines were comparable between the 2 groups. Two trials^[13,14]^ used electro-acupuncture. Two studies^[15,16]^ used ordinary acupuncture. One study^[17]^ used transcutaneous electrical acupoint stimulation. One study^[18]^ used transcutaneous electrical nerve stimulation. Three studies^[19–21]^ used meridian warm compress. One study^[22]^ used wrist-ankle acupuncture. One study^[23]^ used acupoint catgut embedding therapy. For further details of these studies, please refer to the feature table of the included studies. The efficacy evaluation criteria of all studies were essentially the same. The treatment cycle in most studies ranged from 1 to 4 weeks, with each treatment lasting 30 to 60 minutes. All study results were reported using pain-related indicators as the primary outcome measures.

### 3.3. Risk assessment of bias in included studies

**Figure 2.**
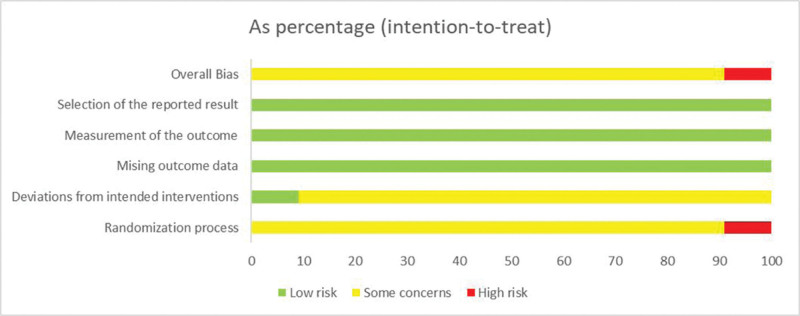
The percentage plot of risk of bias for the included study.

**Figure 3.**
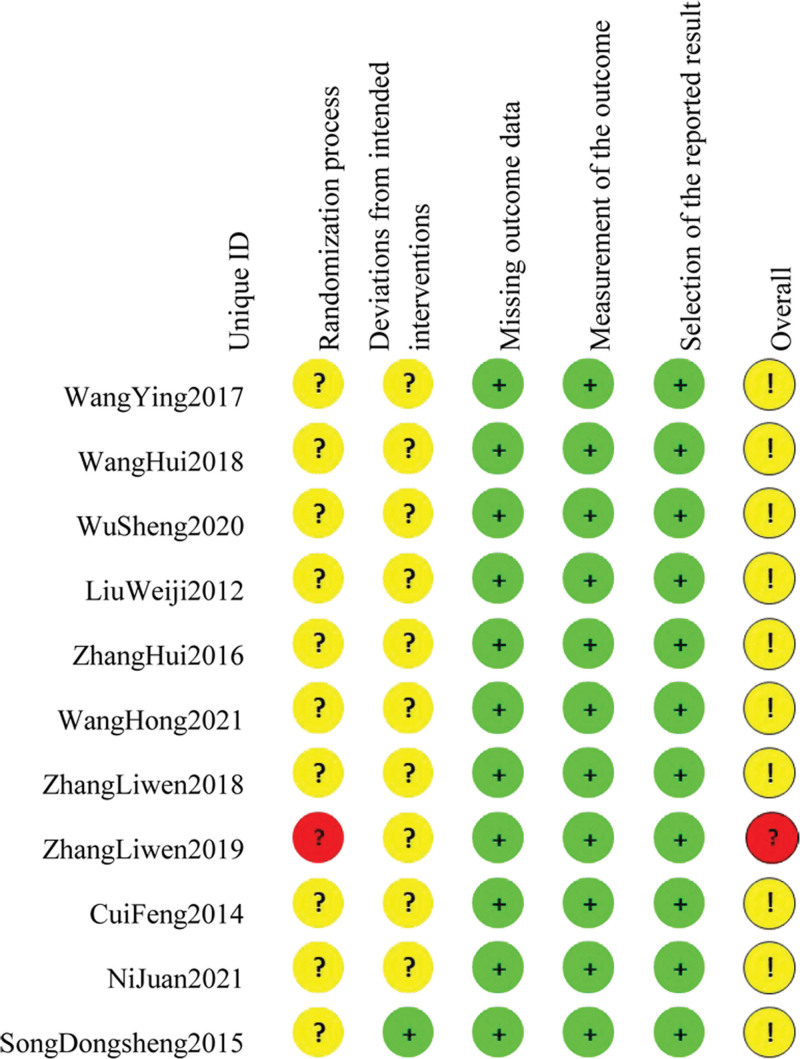
The summary plot of risk of bias for each included study.

We assessed the risk of bias in the included studies using the Rob 2.0 tools. Among the 11 included studies, 4 RCTs^[14,15,18,22]^ used the random number table method; 1 study^[19]^ used the envelope method; 5 articles^[13,16,17,21,23]^ did not mention the method of the specific random grouping; 1 study^[20]^ did not describe the allocation method. None of the studies reported hidden allocation. In the “Randomization process,” 1 study^[20]^ was rated as “high risk,” while the rest were rated as having “some concerns.” None of the literature mentioned blinding, so the “Deviations from intended interventions” was rated as “some concerns.” All literature data are complete, so the “Missing outcome data” was rated as “low risk.” All studies reported outcome measures appropriately, so the “Measurement of the outcome” was rated as “low risk.” All study outcome data were analyzed as scheduled, so the “Selection of the reported result” was rated as “low risk.” Regarding the “Overall Bias,” all the literature was rated as “some concerns,” and the risk of bias assessment of the included literature is shown in Figures 2 and 3.

### 3.4. Meta-analysis results

#### 3.4.1. Pain intensity score

**Figure 4.**
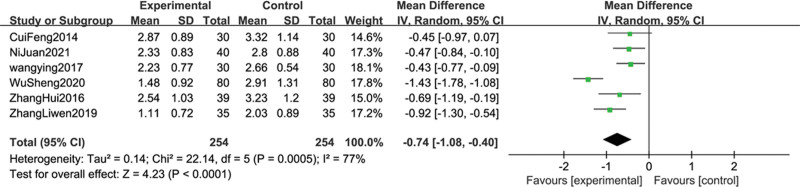
The forest plot of NRS score of acupuncture combined with opioids drugs versus opioids drugs alone for lung cancer-related pain.

A total of 8 studies^[13,15–18,20–22]^ reported pain intensity scores, including the NRS, VAS, and BPI. 6 studies,^[13,15,17,20–22]^ used NRS scoring criteria, 1 study^[18]^ used BPI scoring criteria, and 1 study^[16]^ used VAS scoring criteria. Since it was not possible to conduct a meta-analysis on a single study, only 6 studies that included NRS scores were analyzed. The heterogeneity test (*P* = .0005, I^2^ = 77%) indicated significant heterogeneity, and therefore the random effect model was used. The source of heterogeneity was analyzed. It may be related to the inconsistency of acupuncture intervention measures, the inconsistent dosage and administration methods of opioid analgesics, and the measurement bias caused by the lack of strict blinding in most studies. The results showed that the combined sample size was 508 cases, and the combined effect was statistically significant (MD = −0.74, 95% CI (−1.08, −0.40), Z = 4.23, *P* < .0001). The diamond-shaped small square falls on the left side of the invalid line, which is advantageous for the treatment group, and does not intersect with the invalid line. This indicates that the pain intensity score of the group receiving acupuncture combined with opioid analgesics is lower than that of the group receiving opioid analgesics alone (see Fig. 4).

#### 3.4.2. Effective rate of analgesia

**Figure 5.**
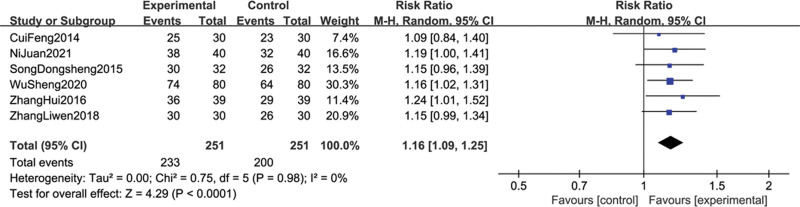
The forest plot of analgesia effective rate of acupuncture combined with opioids drugs versus opioids drugs alone for lung cancer-related pain.

Six studies^[15,17,19,21–23]^ reported the effective rate of analgesia after treatment. In the treatment group, 233 out of 251 patients experienced effective analgesia. In the control group, 200 out of 251 patients experienced effective analgesia. The heterogeneity test (*P* = .98, I^2^ = 0%), indicating that there was small statistical heterogeneity. However, due to the presence of significant clinical heterogeneity, the random effect model was used. The results showed that the combined sample size was 502 cases, and the combined effect size was statistically significant (RR = 1.16, 95% CI [1.09, 1.25], Z = 4.29, *P* < .0001). The diamond-shaped small square falls on the right side of the invalid line, which is advantageous for the treatment group, and does not intersect with the invalid line. This means that the analgesic effect of acupuncture, when combined with opioid analgesics, is superior to that of opioid analgesics alone (see Fig. 5).

#### 3.4.3. Adverse reactions

**Figure 6.**
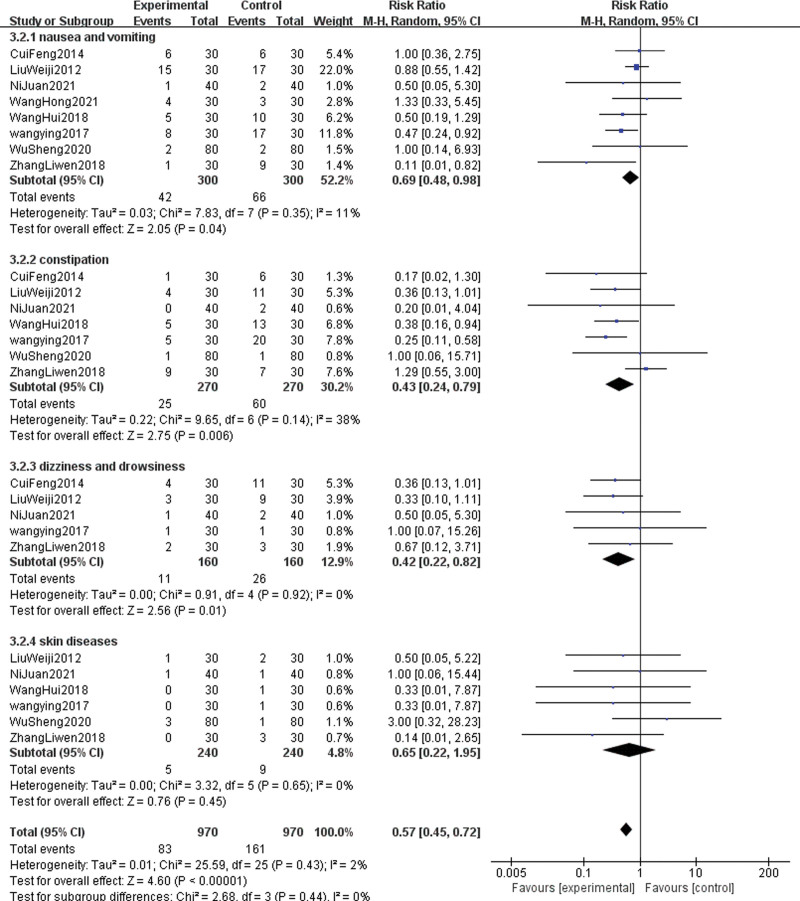
The forest plot of adverse reactions of acupuncture combined with opioids drugs versus opioids drugs alone for lung cancer-related pain.

The adverse reactions reported by the outcome indicators mainly include: nausea and vomiting, constipation, dizziness and drowsiness, and skin diseases (pruritus, rash, and urticaria). Due to the significant clinical heterogeneity, the analysis of adverse effects was performed using the random-effects model. ① Eight articles^[13–16,18,19,21,22]^ reported nausea and vomiting. After conducting a heterogeneity test (*P* = .35, I^2^ = 11%), a random effect model was used. The results showed that the incidence of nausea and vomiting in the experimental group was lower (RR = 0.69, 95% CI [0.48, 0.98], Z = 2.05, *P* = .04). ② Constipation was reported in 7 articles.^[13–16,19,21,22]^ After conducting a heterogeneity test (*P* = .14, I^2^ = 38%), a random effect model was used. The results showed that the incidence of constipation in the experimental group was lower than that in the control group (RR = 0.43, 95% CI [0.24, 0.79], Z = 2.75, *P* = .006). ③ Five articles^[13,16,19,21,22]^ reported dizziness and drowsiness. After conducting a heterogeneity test (*P* = .92, I^2^ = 0 %), a random effect model was used. The results showed that the incidence of dizziness and drowsiness in the group that received acupuncture combined with opioid analgesics was lower than that in the control group (RR = 0.42, 95% CI [0.22, 0.82], Z = 2.56, *P* = .01). ④ Six studies^[13–16,19,22]^ reported skin diseases such as pruritus, rash, and urticaria. After conducting a heterogeneity test (*P* = .65, I^2^ = 0 %), a random effect model was used. The results showed that there was insufficient evidence to prove a difference in the incidence of skin diseases between acupuncture combined with opioid analgesics and opioid analgesics alone (RR = 0.65, 95% CI [0.22, 1.95], Z = 0.76, *P* = .45). The combined effect was statistically significant (RR = 0.57, 95% CI [0.45, 0.72], Z = 4.60, *P* < .00001). The diamond-shaped small square falls on the left side of the invalid line, which is beneficial to the treatment group, and does not intersect with the invalid line. This indicates that the incidence of adverse reactions in the acupuncture combined with opioid analgesic group is lower than that in the opioid analgesic group (see Fig. 6).

### 3.5. Quality of evidence for the GRADE system rating

We assessed the quality of evidence for each outcome measure using GRADEpro and determined that the 3 outcome measures had low-quality evidence. A summary findings table is provided (see Table 2).

**Table 2:** Summary table of findings of acupuncture combined with opioid for lung cancer-related pain.

Outcomes of acupuncture combined with opioid for lung cancer related pain					
Patient or population: patients with lung cancer related pain Intervention: acupuncture combined with opioid Control: opioid					
Outcomes	Risk assessment (95% CI)		Relative effect (95% CI)	Number of participants (studies)	Quality of evidence (GRADE)
	Control risk (per 1000 people)	Intervention risk (per 1000 people)			
Pain intensity score		The mean pain intensity score in the intervention groups was 0.74 lower (1.08–0.4 lower)		508 (6 studies)	⊕⊕⊖⊖Low*^,^†
Effective rate of analgesia	797	924 (869–996)	RR 1.16 (1.09–1.25)	502 (6 studies)	⊕⊕⊖⊖Low*^,^†
Adverse reactions: nausea and vomiting	220	141 (101–194)	RR 0.64 (0.46–0.88)	600 (8 studies)	⊕⊕⊖⊖Low*^,^†
Adverse reactions: constipation	222	93 (62–140)	RR 0.42 (0.28–0.63)	540 (7 studies)	⊕⊕⊖⊖Low*^,^†
Adverse reaction: dizziness and drowsiness	162	68 (36–132)	RR 0.42 (0.22–0.81)	320 (5 studies)	⊕⊕⊖⊖Low*^,^†
Adverse reactions: skin diseases	38	23 (9–61)	RR 0.62 (0.24–1.62)	480 (6 studies)	⊕⊕⊖⊖Low*^,^†

Control risk were based on the median risk of each study control group.

Intervention risk (and its 95% CI) are based on the control risk in the control group and the relative effects of the intervention (and its 95% CI).

CI: confidence interval; RR: Risk ratio; GRADE: working group grades of evidence; High quality: further research is very unlikely to change our confidence in the estimate of effect; Moderate quality: further research is likely to have an important impact on our confidence in the estimate of effect and may change the estimate; Low quality: further research is very likely to have an important impact on our confidence in the estimate of effect and is likely to change the estimate; Very low quality: very uncertain about the estimate.

* The implementation of randomization or blinding carries a risk of bias.

† CI range is narrow or no overlap.

### 3.6. Sensitivity analysis and publication bias detection

In this study, the sensitivity analysis was conducted using the exclusion method to systematically exclude each study one by one. The results revealed no significant changes in MD and confidence intervals for pain intensity score, and no significant changes in RR and confidence intervals for the effective rate of analgesia and adverse reactions. This indicates that the analytical results of this study have a certain stability. Due to the inclusion of fewer than 20 articles, funnel plots are not applicable for evaluating publication bias. Therefore, the study may have potential publication bias.

### 3.7. Analysis of acupoint frequency

**Figure 7.**
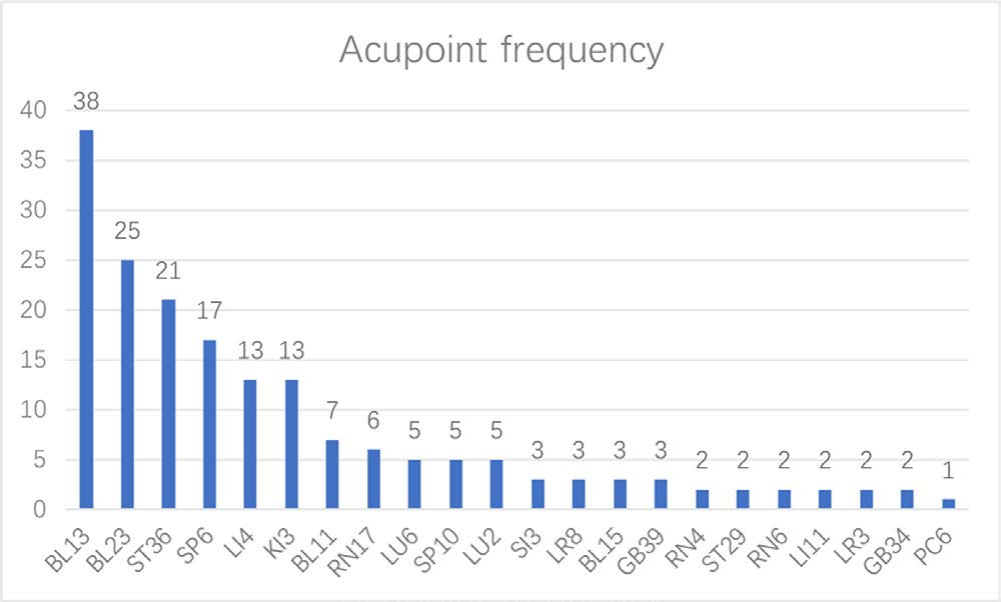
Frequency histogram of acupoints used in the treatment of lung cancer-related pain.

A total of 22 acupoints were included, with a combined frequency of 180 occurrences. The top 10 acupoints were used with a frequency of more than 5 times, constituting 83.33% of the total frequency. Among them, the top 5 acupoints were BL13, BL23, ST36, SP6, and LI4. The frequency of the aforementioned 5 acupoints accounted for 63.33% of the total frequency (see Fig. 7).

### 3.8. Associative rule analysis

**Figure 8.**
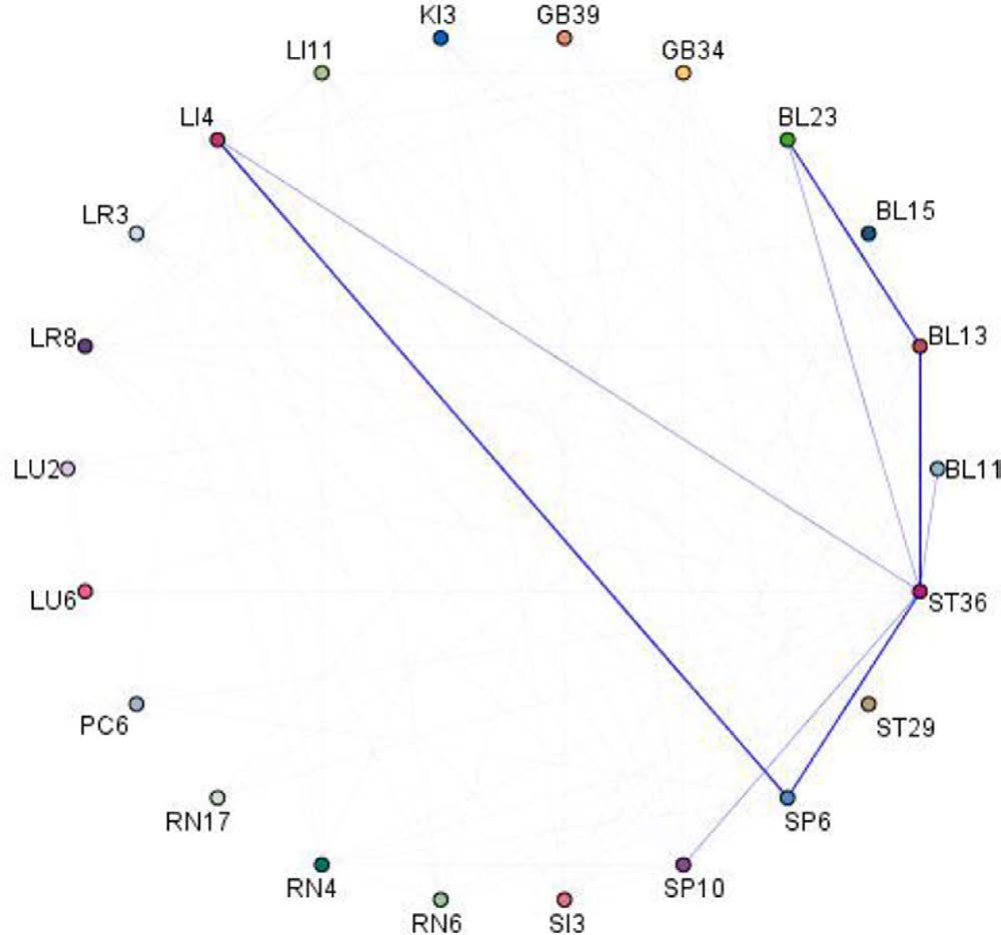
The association rule complex network graph of acupoints for the treatment of lung cancer-related pain.

The complex network graph was created using SPSS Modeler 18.0 software. A total of 8 association rules were generated, and 4 strong association rules of acupoints were obtained. These include LI4-SP6, SP6-ST36, BL13-ST36, and BL13-BL23 (see Fig. 8).

## 4. Discussion

The incidence and mortality of lung cancer remain high globally and are expected to increase, particularly in developing countries.^[24]^ The research data shows that more lung cancer patients will experience pain related to their cancer in the future. The commonly used three-step analgesic therapy is no longer sufficient to meet the needs of patients. We need new and improved treatment strategies to alleviate cancer pain.

In the early stages, there were some studies on acupuncture treatment for cancer pain.^[25–27]^ They were similar to this study in terms of treatment measures, focusing on acupuncture as an adjuvant treatment for cancer pain. However, there were also some differences. Previous studies did not limit the specific type of cancer for patients with cancer pain, making the sample size too broad and not targeted. In this study, we specifically examined lung cancer patients, resulting in more accurate findings. Additionally, the database we searched is more comprehensive and up-to-date, setting it apart from previous studies.

As an adjuvant therapy for relieving cancer pain, acupuncture can serve as an analgesic for various types, stages, and degrees of cancer pain.^[28,29]^ Acupuncture can also alleviate cancer treatment-related side effects, such as fatigue, insomnia, chemotherapy-related dyspepsia syndrome, xerostomia, anxiety, and depression.^[30]^ This can improve the quality of life and prolong the life cycle. According to the theory of traditional Chinese medicine, “pain is caused by obstruction.” Acupuncture plays an analgesic role by stimulating acupoints and clearing blockages in the meridians throughout the body. Contemporary research reveals that the stimulation of acupuncture on acupoints can be transmitted to the relevant regions of the spinal cord and brain. On one hand, it can inhibit the activation of spinal glial cells to reduce pain. On the other hand, by stimulating different acupoints, it can change the functional connection and neuronal activity of the corresponding brain region, resulting in an analgesic effect.^[31,32]^At the same time, acupuncture points can directly activate subcutaneous mast cells, leading to the production of analgesic effects.^[33]^ These studies show that acupuncture not only has a local analgesic effect, but also can regulate the brain distally by stimulating acupoints to produce an analgesic effect. Although the mechanism of acupuncture has not yet been clarified, the evidence accumulated from clinical trials shows that acupuncture is a safe and effective adjunct therapy for chronic cancer pain.

High-frequency acupoints can reflect the primary acupoints utilized in the current clinical acupuncture treatment of lung cancer-related pain to some extent. In our study, we found that the top 5 acupoints with the highest frequency of use are: BL13, BL23, ST36, SP6, and LI4. Feishu (BL13) and Shenshu (BL23) belong to the Bladder Meridian of Foot-Taiyang. Acupoints are specific points where the qi of zang-fu organs is infused into the back, exerting a beneficial regulatory effect on the lungs. Traditional Chinese medicine believes that the lungs and kidneys are closely related, physiologically dependent on each other, and pathologically influenced by each other. Modern research has found that acupuncture treatment of BL13 and BL23 can protect pulmonary function by reducing cellular inflammation and the production of IL-8 and TNF-α.^[34]^ Scar moxibustion at Zusanli (ST36) and BL13 can improve the quality of life of patients with non-small-cell lung cancer.^[35]^ Zusanli (ST36) belong to the Stomach Meridian of Foot-Yangming, serves as a focal point for the convergence of qi and blood in the human body. It plays a crucial role in regulating the meridians and replenishing qi and blood. Modern research indicates that moxibustion on ST36 can promote gastric mucosal apoptosis and delay the progression of gastric cancer.^[36]^ Sanyinjiao (SP6) belong to the Spleen Meridian of Foot-Taiyin, is the intersection point of the 3 yin meridians of the foot. Modern studies have shown that electro-acupuncture stimulation of ST36 and SP6 can reduce mice’s tolerance to morphine, making it suitable for treating patients with opioid tolerance.^[37]^ Hegu (LI4) is the source point of the Large Intestine Meridian of Hand-Yangming. It can adjust the circulation of qi and blood throughout the body. Modern studies have shown that the anatomical position of LI4 is close to the vascular branch of the superficial branch of the radial nerve. Therefore, stimulating LI4 can help treat peripheral circulation failure.^[38]^ In addition, studies have proved that acupuncture stimulation of LI4, ST36, and SP6 can effectively relieve cancer pain.^[39]^

The results of the meta-analysis showed that the effectiveness rate of analgesia was higher when acupuncture was combined with opioid analgesics compared to using traditional opioid analgesics alone. Additionally, the combination therapy resulted in lower pain intensity scores and a lower incidence of adverse reactions.

There are several limitations to this study. The quality of the clinical randomized controlled trials included was low, and there was a large degree of clinical heterogeneity. The literature included in this study primarily originates from China and lacks robust research evidence from around the world. During the systematic review process, there may be limitations such as inaccuracies in evaluating the quality of the included literature. Additionally, many studies have not been registered for clinical trials, which compromises the transparency and traceability of the study. It is also challenging to access unpublished studies, leading to a potential risk of publication bias. Publication bias may lead to the exaggeration or underestimation of research results, thus affecting the conclusions of the meta-analysis.

## 5. Conclusion

Our study demonstrates that the combination of acupuncture and opioid analgesics is more effective in treating lung cancer-related pain compared to using opioid analgesics alone. This finding can help alleviate the pain of patients with lung cancer and provide valuable references for clinicians and researchers. To expand the application of acupuncture in cancer-related pain, it is essential to enhance research in the field of acupuncture treatment for cancer pain in the future. Firstly, conduct more large-scale, multi-center, high-quality original research. Secondly, focus on conducting basic discipline research on the mechanism of acupuncture. Thirdly, further research is needed on acupuncture to delay the development of malignant tumors.

## Author contributions

**Conceptualization:** Liting Jia, Shuquan Chen.

**Methodology:** Liting Jia, Keyi Wang.

**Supervision:** Shuquan Chen.

## Supplementary Material


