# Supplementary material for: Acupuncture combined with opioid for treatment of lung cancer-related pain: A systematic review and meta-analysis
Source: Medicine (Baltimore). 2024 Oct 18;103(42):e40158. doi: 10.1097/MD.0000000000040158 (PMC11495702; doi:10.1097/MD.0000000000040158)
Supplement: Supplementary file 1 [file medi-103-e40158-s001.docx]

**Supplementary material S1： Literature retrieval strategy**

The search time ranged from inception to february 2023.

We have included the complete search strategy used in the 8 databases retrieved by this study in the supplementary material.

### PubMed 、The Cochrane Library search strategy

#1MeSH descriptor: [Lung Neoplasms] explode all trees

#2lung cancer* [Title/Abstract]

#3lung carcinoma*[Title/Abstract]

#4lung malignan* [Title/Abstract]

#5lung neoplasm*[Title/Abstract]

#6lung tumo*[Title/Abstract]

#7pulmonary cancer*[Title/Abstract]

#8pulmonary carcinom*[Title/Abstract]

#9pulmonary malignan*[Title/Abstract]

#10pulmonary neoplasm*[Title/Abstract]

#11pulmonary tumo*[Title/Abstract]

#12MeSH descriptor: [Carcinoma, Non‐Small‐Cell Lung] explode all trees

#13nonsmall cell lung cancer*[Title/Abstract]

#14non small cell lung cancer*[Title/Abstract]

#15nonsmall cell lung carcinoma*[Title/Abstract]

#16non small cell lung carcinoma*[Title/Abstract]

#17NSCLC[Title/Abstract]

#18MeSH descriptor: [Carcinoma, Small Cell] explode all trees

#19oat cell carcinoma*[Title/Abstract]

#20oat cell lung carcinoma*[Title/Abstract]

#21oat cell lung cancer*[Title/Abstract]

#22oat cell cancer*[Title/Abstract]

#23SCLC[Title/Abstract]

#24small cell lung cancer*[Title/Abstract]

#25small cell lung carcinom*[Title/Abstract]

#26 #1 or #2 or #3 or #4 or #5 or #6 or #7 or #8 or #9 or #10 or #11 or #12 or #13 or #14 or #15 or #16 or #17 or #18 or #19 or #20 or #21 or #22 or #23 or #24 or #25

#27 MeSH descriptor:[Pain]explode all trees

#28 Pain*[Title/Abstract]

#29 #27 or #28

#30 MeSH descriptor:[Acupuncture Therapy]explode all trees

#31(Acupuncture Treatment or acupuncture$ or acupressure or acupuncture needle* or acup*).[Title/Abstract]

#32(acupuncture analgesia or acupuncture anesthesia ).[Title/Abstract]

#33(electroacupuncture or electro acupuncture or electro‐acupuncture)[Title/Abstract]

#34(auricular acupuncture or ear acupuncture ).[Title/Abstract]

#35(fire needle$ or warming needle$).[Title/Abstract]

#36moxibustion.[Title/Abstract]

#37(meridian$ or Jing Luo or jingluo).[Title/Abstract]

#38(acupoint$ or acu-point$ or acupuncture point$).[Title/Abstract]

#39 #30 or #31 or #32 or #33 or #34 or #35 or #36 or #37 or #38

#40 MeSH descriptor:[narcotics]explode all trees

#41 Analgesics, Opioid [Title/Abstract]

#42(morphine or buprenorphine or codeine or dextromoramide or diphenoxylate or dipipanone or dextropropoxyphene or propoxyphene or diamorphine or dihydrocodeine or alfentanil or fentanyl or remifentanil or meptazinol or methadone or nalbuphine or oxycodone or papaveretum or pentazocine or meperidine or pethidine or phenazocine or hydrocodone or hydromorphone or levorphanol or oxymorphone or butorphanol or dezocine or sufentanil or ketobemidone).[Title/Abstract]

#43 #40 or #41 or #42

#44 #26 and #29 and #39 and #43

### Web of Science search strategy

#1 TS=（lung neoplasm* or lung cancer* or lung carcinoma* or lung malignan* or lung tumo* or pulmonary cancer* or pulmonary carcinom* or pulmonary malignan* or pulmonary neoplasm* or pulmonary tumo* or nonsmall cell lung cancer* or non small cell lung cancer* or nonsmall cell lung carcinoma* or non small cell lung carcinoma* or NSCLC or oat cell carcinoma* or oat cell lung carcinoma* or oat cell lung cancer* or oat cell cancer* or SCLC or small cell lung cancer* or small cell lung carcinom*）

#2 TS=（Pain*）

#3 TS=（Acupuncture Therapy or Acupuncture Treatment or acupuncture$ or acupressure or acupuncture needle* or acup* or acupuncture analgesia or acupuncture anesthesia or electroacupuncture or electro acupuncture or electro‐acupuncture or auricular acupuncture or ear acupuncture or fire needle$ or warming needle$ or moxibustion or meridian$ or Jing Luo or jingluo or acupoint$ or acu-point$ or acupuncture point$）

#4 TS=（narcotics or Analgesics, Opioid or morphine or buprenorphine or codeine or dextromoramide or diphenoxylate or dipipanone or dextropropoxyphene or propoxyphene or diamorphine or dihydrocodeine or alfentanil or fentanyl or remifentanil or meptazinol or methadone or nalbuphine or oxycodone or papaveretum or pentazocine or meperidine or pethidine or phenazocine or hydrocodone or hydromorphone or levorphanol or oxymorphone or butorphanol or dezocine or sufentanil or ketobemidone）

#5 #1 and #2 and #3 and #4

### Embase search strategy

#1 'lung tumor'/exp OR 'lung cancer*':ab,ti OR 'lung carcinoma*':ab,ti OR 'lung malignan*':ab,ti OR 'lung neoplasm*':ab,ti OR 'lung tumo*':ab,ti OR 'pulmonary cancer*':ab,ti OR 'pulmonary carcinom*':ab,ti OR 'pulmonary malignan*':ab,ti OR 'pulmonary neoplasm*':ab,ti OR 'pulmonary tumo*':ab,ti OR 'lung non small cell cancer'/exp OR 'nonsmall cell lung cancer*':ab,ti OR 'non small cell lung cancer*':ab,ti OR 'nonsmall cell lung carcinoma*':ab,ti OR 'non small cell lung carcinoma*':ab,ti OR 'nsclc':ab,ti OR 'small cell lung cancer'/exp OR 'oat cell carcinoma*':ti,ab OR 'oat cell lung carcinoma*':ti,ab OR 'oat cell lung cancer*':ti,ab OR 'oat cell cancer*':ti,ab OR 'sclc':ti,ab OR 'small cell lung cancer*':ti,ab OR 'small cell lung carcinom*':ti,ab

#2 'pain*'/exp OR 'pain*':ab,ti

#3 'Acupuncture Therapy'/exp OR ‘Acupuncture Treatment’:ab,ti OR acupuncture$:ab,ti OR acupressure:ab,ti OR ‘acupuncture needle*’:ab,ti OR acup*:ab,ti OR ‘acupuncture analgesia’:ab,ti OR ‘acupuncture anesthesia’:ab,ti OR Electroacupuncture:ab,ti OR ‘electro acupuncture’:ab,ti OR ‘electro‐acupuncture’:ab,ti OR ‘auricular acupuncture’:ab,ti OR ‘ear acupuncture’:ab,ti OR ‘fire needle$’:ab,ti OR ‘warming needle$’:ab,ti OR moxibustion:ab,ti OR meridian$:ab,ti OR ‘Jing Luo’:ab,ti OR jingluo:ab,ti OR acupoint$:ab,ti OR ‘acupuncture point$’:ab,ti

#4 'narcotics'/exp OR 'Analgesics, Opioid'/exp OR Morphine:ab,ti OR buprenorphine:ab,ti OR codeine:ab,ti OR dextromoramide:ab,ti OR diphenoxylate:ab,ti OR dipipanone:ab,ti OR dextropropoxyphene:ab,ti OR propoxyphene:ab,ti OR diamorphine:ab,ti ORdihydrocodeine:ab,ti OR alfentanil:ab,ti OR fentanyl:ab,ti OR remifentanil:ab,ti OR meptazinol:ab,ti OR methadone:ab,ti OR nalbuphine:ab,ti OR oxycodone:ab,ti OR papaveretum:ab,ti OR pentazocine:ab,ti OR meperidine:ab,ti OR pethidine:ab,ti OR phenazocine:ab,ti OR hydrocodone:ab,ti OR hydromorphone:ab,ti OR levorphanol:ab,ti OR oxymorphone:ab,ti OR butorphanol:ab,ti OR dezocine:ab,ti OR sufentanil:ab,ti OR ketobemidone:ab,ti

#5 #1 and #2 and #3 and #4

**Chinese database retrieval strategy**

#1 The descriptor：Lung Neoplasms

#2 Text/key word/abstract：Lung Neoplasms + lung cance + lung carcinoma + lung malignan + nonsmall cell lung cancer + small cell lung cancer

#3 #1 OR #3

#4 The descriptor：pain

#5 Text/key word/abstract：Pain + pain syndrome

#6 #4 OR #5

#7 The descriptor：Acupuncture Therapy

#8 Text/key word/abstract：Acupuncture Therapy+Acupuncture Treatment+acupuncture analgesia+acupuncture anesthesia+electroacupuncture+auricular acupuncture+fire needle+moxibustion+meridian+acupoint+transcutaneous electrical acupoint stimulation+ electrophotoluminescence +ear acupoint +Ear hole pressure beans+point injection therapy+Acupoint catgut embedding + acupoint application

#9 #7 OR #8

#10 The descriptor：Analgesics, Opioid

#11 Text/key word/abstract：Analgesics, Opioid+morphine + buprenorphine + codeine + dextromoramide + diphenoxylate + dipipanone + dextropropoxyphene + propoxyphene +diamorphine + dihydrocodeine + alfentanil + fentanyl + remifentanil + meptazinol + methadone +nalbuphine + oxycodone + papaveretum + pentazocine +meperidine + pethidine + phenazocine + hydrocodone + hydromorphone + levorphanol + oxymorphone +butorphanol + dezocine + sufentanil+ ketobemidone

#12 #10 OR #11

#13 #3 AND #6 AND #9 AND #12
